# High-Throughput Measure of Mitochondrial Superoxide Levels as a Marker of Coronary Artery Disease to Accelerate Drug Translation in Patient-Derived Endothelial Cells Using Opera Phenix^®^ Technology

**DOI:** 10.3390/ijms25010022

**Published:** 2023-12-19

**Authors:** Weiqian E. Lee, Marie Besnier, Elijah Genetzakis, Owen Tang, Katharine A. Kott, Stephen T. Vernon, Michael P. Gray, Stuart M. Grieve, Michael Kassiou, Gemma A. Figtree

**Affiliations:** Kolling Institute, University of Sydney, Sydney, NSW 2006, Australiamichael.kassiou@sydney.edu.au (M.K.)

**Keywords:** mitochondria, high-content imaging, high-throughput screening, endothelial dysfunction, endothelial colony-forming cells, drug screening, mROS

## Abstract

Improved human-relevant preclinical models of coronary artery disease (CAD) are needed to improve translational research and drug discovery. Mitochondrial dysfunction and associated oxidative stress contribute to endothelial dysfunction and are a significant factor in the development and progression of CAD. Endothelial colony-forming cells (ECFCs) can be derived from peripheral blood mononuclear cells (PBMCs) and offer a unique potentially personalised means for investigating new potential therapies targeting important components of vascular function. We describe the application of the high-throughput and confocal Opera Phenix^®^ High-Content Screening System to examine mitochondrial superoxide (mROS) levels, mitochondrial membrane potential, and mitochondrial area in both established cell lines and patient-derived ECFCs simultaneously. Unlike traditional plate readers, the Opera Phenix^®^ is an imaging system that integrates automated confocal microscopy, precise fluorescent detection, and multi-parameter algorithms to visualize and precisely quantify targeted biological processes at a cellular level. In this study, we measured mROS production in human umbilical vein endothelial cells (HUVECs) and patient-derived ECFCs using the mROS production probe, MitoSOX^TM^ Red. HUVECs exposed to oxidized low-density lipoprotein (oxLDL) increased mROS levels by 47.7% (*p* < 0.0001). A pooled group of patient-derived ECFCs from participants with CAD (*n* = 14) exhibited 30.9% higher mROS levels compared to patients with no CAD when stimulated with oxLDL (*n* = 14; *p* < 0.05). When tested against a small group of candidate compounds, this signal was attenuated by PKT-100 (36.22% reduction, *p* = 0.03), a novel P2X7 receptor antagonist. This suggests the P2X7 receptor as a valid target against excess mROS levels. As such, these findings highlight the potential of the MitoSOX-Opera Phenix technique to be used for drug discovery efforts in CAD.

## 1. Introduction

High-throughput screening (HTS) plays a pivotal role in drug discovery research by facilitating the rapid evaluation of a vast number of target compounds. Traditionally, microplate readers have been widely used to assess compound libraries against purified target proteins. While this approach has proven to be efficient, it falls short in capturing the intricate cell-to-cell variations, interactions, and phenotypic nuances that are essential for a comprehensive understanding of complex biological systems as well as opportunities for personalised medicine during translational research.

Coronary artery disease (CAD) is a common clinically significant cardiovascular condition responsible for a plurality of deaths globally [1]. The most common form of CAD is characterised by the progressive development of atherosclerotic plaque within the walls of coronary arteries [2]. This intricate process can lead to a gradual narrowing of the lumen of the artery and ischaemia related to reduced blood flow to the heart muscle [2]. Unstable atherosclerotic plaque can be susceptible to erosion or rupture, activating a coagulation cascade and leading to acute myocardial infarction or heart attack [2]. The structural and functional integrity of the endothelium is a critical regulator of atherosclerotic initiation. This is believed to begin with a deposit of lipid species, in particular oxidised low-density lipoprotein (oxLDL) cholesterol [3]. The oxLDL accumulates in the tunica intima of the coronary vessels which causes endothelial dysfunction, triggering an immune-mediated response and cascade of events that ultimately leads to the formation of atherosclerotic plaque [3,4,5,6,7,8] and development of high-risk plaque features predisposed to rupture and myocardial infarction [7]. The mitochondria, a primary source of reactive oxygen species (ROS) production, play a fundamental role in this process. Previously, mitochondrial dysfunction and excessive ROS production have been associated with the development of atherosclerosis amongst other cardiovascular diseases [3,9,10]. Thus, the protection of mitochondria from dysregulation under otherwise pro-atherogenic conditions presents a promising avenue for promoting more normal vascular homeostasis. However, there has been limited progress due to poor results from mitochondria-targeting agents against patients with cardiovascular disorders and current limitations in measuring dysregulated redox signalling [3,10].

Previously, we demonstrated the utility of patient-derived endothelial colony-forming cells (ECFCs), derived from peripheral blood mononuclear cells (PBMCs), as a valuable model for assessing endothelial dysfunction directly relevant to human disease [11]. Notably, we have demonstrated a strong correlation between mitochondrial superoxide (mROS) production and the severity of CAD as measured using the cardiac CT-derived coronary artery calcium score (CACS) or Gensini score [11].

**Figure 1:**
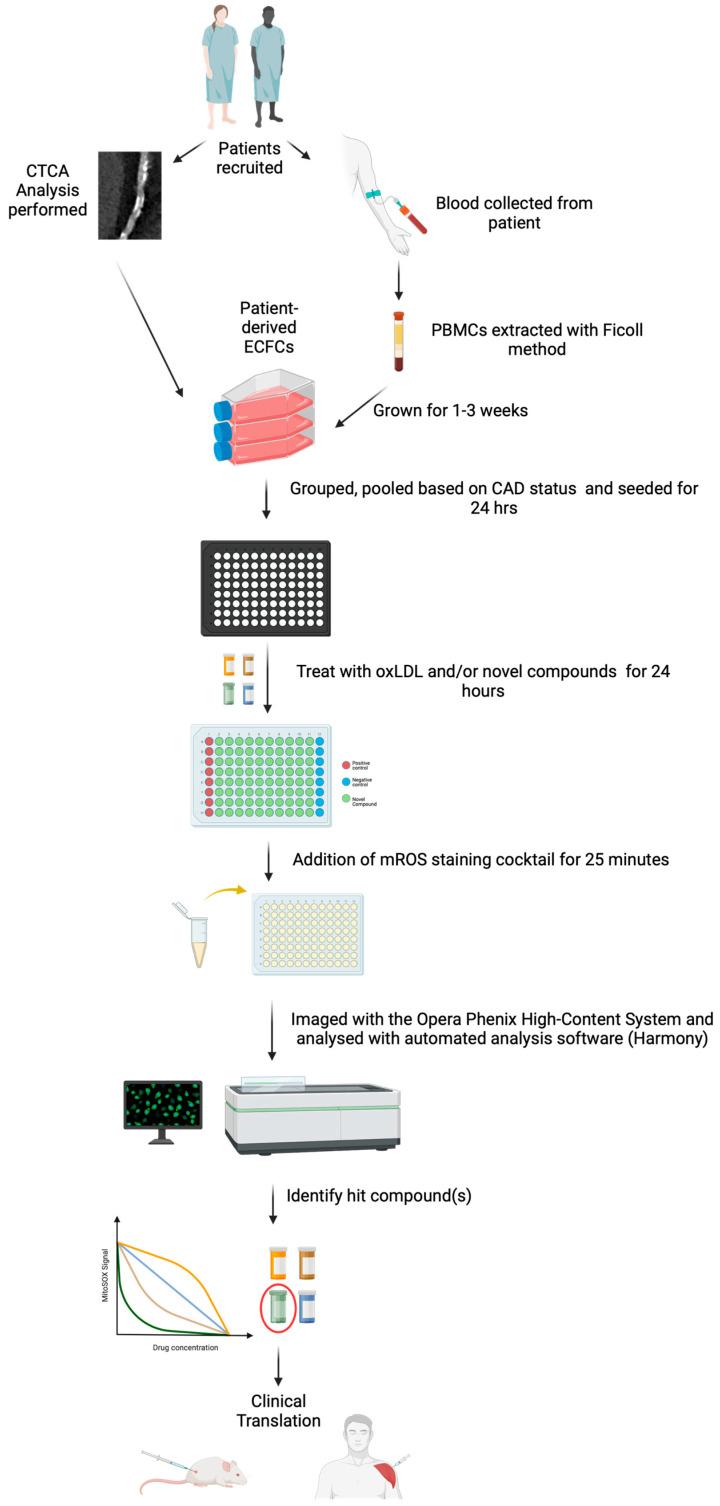
Schematic diagram of the workflow of the approach for diagnostic and therapeutic application of the Opera Phenix^®^ High-Content System. Created using BioRender.com (accessed 12 October 2023).

Here, we expand upon this by applying a high-throughout, sensitive, confocal platform, the Opera Phenix^®^ High-Content Screening System, to patient-derived ECFCs. This system is multiplexed with MitoSOX^TM^ Red and MitoTracker^TM^ Deep Red to efficiently analyse mROS levels, mitochondrial membrane potential, and mitochondrial area simultaneously under cell culture conditions. This overcomes several issues related to the use of MitoSOX^TM^ in microplate readers whereby the fluorescence of the MitoSOX^TM^ signal generated can be affected by mitochondrial membrane potential, mitochondrial size, as well as non-specific signals generated from non-mitochondrial sources [12]. Furthermore, we study and highlight two potential proof-of-concepts of this platform, including (1) its use as a diagnostic tool for identifying risk for CAD significant for age and sex; and (2) as a platform to evaluate the effectiveness of novel drugs against excess mROS levels (Figure 1). The use of a highly sensitive high-throughput platform for measuring mitochondrial function in CAD-positive patient-derived ECFCs overcomes several of the unique challenges relevant to atherosclerotic CAD, where tissue samples are largely unavailable. This is particularly relevant given that humans are almost unique in their susceptibility to atherosclerotic CAD and spontaneous myocardial infarction [13].

## 2. Results

### 2.1. Evaluation of MitoSOX^TM^ Red Signal after oxLDL Stimulation in HUVECs Using a Traditional Microplate Reader

**Figure 2:**
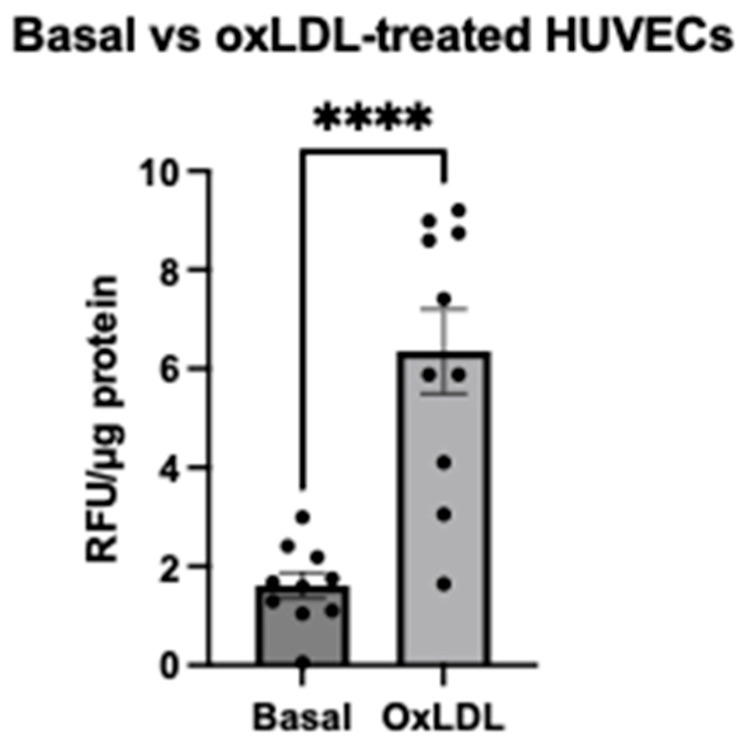
Evaluation of fluorescent microscopy results of mROS production of HUVECs stimulated with or without oxLDL. MROS^•−^ was evaluated in HUVECs using 5 μM MitoSOX^TM^ Red. Relative fluorescence was normalised to protein content (per μg). Data were represented as mean ± S.E.M. Ten independent experiments were performed (*n* = 10). Each condition was performed in triplicate. **** *p* < 0.0001, paired samples *t*-test was performed.

We first examined the effect of oxLDL stimulation on mROS production in the commercially available human umbilical vein endothelial cells (HUVECs) using a traditional microplate reader. After loading with MitoSOX^TM^ Red for 25 min, basal levels of mROS were measured at 1.61 relative fluorescence unit (RFU) per μg of protein in HUVECS. After oxLDL stimulation, mROS levels were increased by four-fold (6.35 RFU/μg protein) (mean difference: 4.74 RFU/μg protein, CI: 3.17 to 6.31, *p* < 0.0001, *n* = 10) (Figure 2). It is notable that this measurement was not fully mitochondria-specific and included the detection of non-specific fluorescence from the well. Background fluorescence was accounted for using empty wells containing no cells to perform background subtraction.

### 2.2. Evaluation of mROS Levels, Mitochondrial Membrane Potential, and Mitochondrial Size after oxLDL Stimulation Using the Opera Phenix

**Figure 3:**
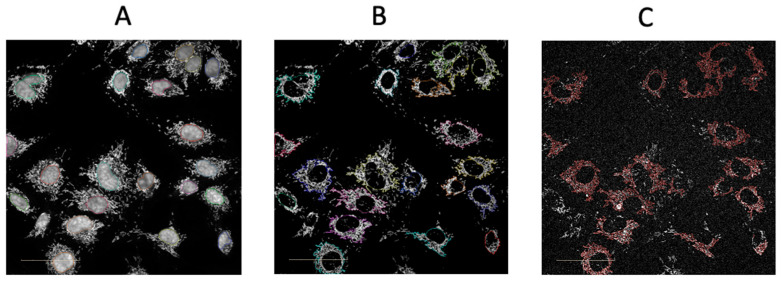
Post hoc automated analysis workflow using the Harmony^®^ High-Content Imaging and Analysis Software (version 5.2). (**A**) The nuclei are first identified by the system using the Hoechst 33342 channel. (**B**) The mitochondrial region is then identified using the MitoTracker^TM^ Deep Red channel around the nuclei that was identified previously. (**C**) MitoSOX^TM^ Red signal captured within the mitochondrial region and MitoSOX^TM^ Red fluorescence is measured using the ‘sliding parabola’ algorithm. Scale bar = 50 μm.

**Figure 4:**
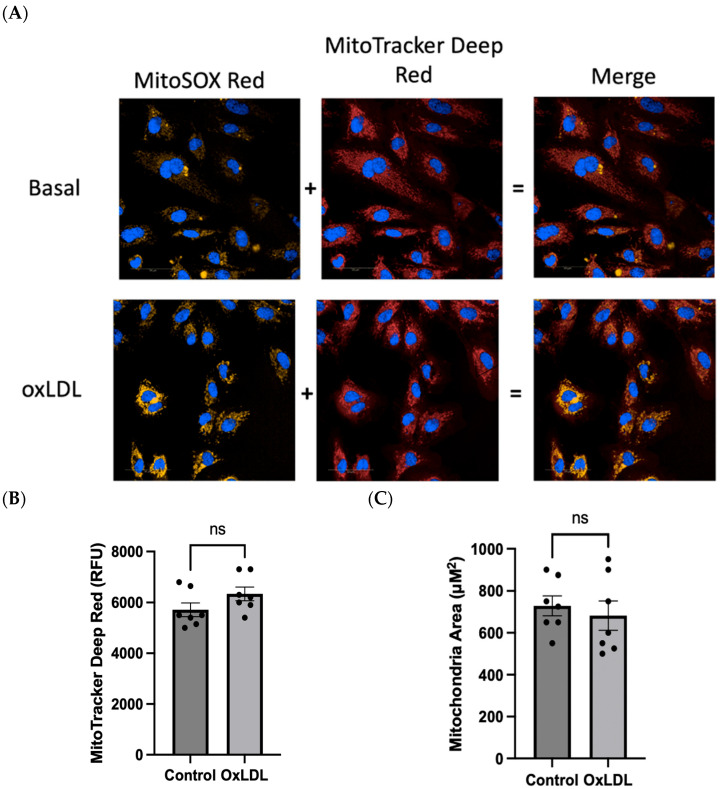
(**A**) Representative Opera Phenix^®^ fluorescent confocal microscopy images showing specific colocalization of mROS production of HUVECs with/without oxLDL stimulation using 5 μM MitoSOX^TM^ Red (yellow), 20 nM MitoTracker^TM^ Deep Red staining (red) of the mitochondrial region of cells, and Hoechst 33342 (blue) to determine cell nuclei. Twenty fields of view were taken per well. Scale bar = 50 μm. Non-specific fluorescence that does not colocalise with the MitoTracker^TM^ Deep Red fluorescence is excluded from the quantification. Evaluation of (**B**) mitochondrial membrane potential and (**C**) mitochondrial area using 20 nM MitoTracker^TM^ Deep Red between HUVECs treated with/without oxLDL using MitoTracker^TM^ Deep Red. Data were represented as mean ± S.E.M. Seven independent experiments were performed. Each condition was performed in triplicate. ns means non-significant, paired samples *t*-test was performed.

We next sought to investigate whether the confocal system of the Opera Phenix^®^ could detect differences in the accumulation of the mitochondria-specific MitoSOX^TM^ Red signal between HUVECs at basal or after oxLDL activation. Furthermore, we also harnessed the confocal system of the Opera Phenix^®^ to use the signal generated from the MitoTracker^TM^ Deep Red to measure membrane potential [14,15,16,17] as well as the size of the mitochondria. Utilising the Opera Phenix^®^ confocal system post-acquisition algorithm, Harmony^®^ (PerkinElmer, Waltham, MA, USA, version 5.2) only the MitoSOX^TM^ Red (in yellow) signal that overlapped with the MitoTracker^TM^ Deep Red (identifies mitochondria, in red) was captured (Figure 3). OxLDL was shown to increase MitoSOX^TM^ Red mitochondria-specific intensity when excited by the 561 nm laser compared to basal (Figure 4A). Furthermore, no significant differences in membrane potential or mitochondrial size were found using MitoTracker Deep Red (Figure 4B,C).

### 2.3. Evaluation of Image Analysis Methods Using Harmony^TM^ (Background Subtraction vs. Rolling Parabola Method)

**Figure 5:**
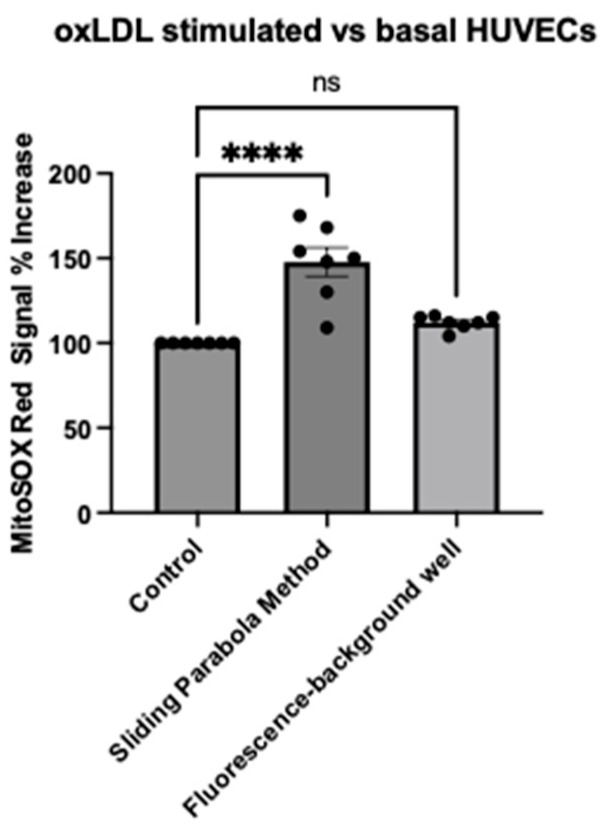
Evaluation of image analysis methods using the automated Harmony^®^ software. The MitoSOX^TM^ Red intensity was evaluated using two methods: ‘sliding parabola’ and ‘fluorescence minus background well’ after 24 h of treatment with or without oxLDL. Data are represented as mean ± S.E.M. Seven independent experiments were performed. Each condition was performed in triplicate. **** *p* < 0.0001, ns means non-significant, one-way ANOVA followed by Dunnett’s multiple comparisons test.

To understand whether quantitative differences observed in the confocal images could be detected using an automated image analysis, we tested two workflows using the Harmony^®^ automated software (version 5.2): the ‘Sliding Parabola Method’ and the ‘fluorescence minus background well’ method. Here, the sliding parabola method was shown to detect differences between the mitochondria-specific MitoSOX^TM^ Red signal in oxLDL-stimulated and control HUVECs (mean difference: 47.7%, CI: 30.9% to 64.58%, *p* < 0.0001, *n* = 7) (Figure 5). No significant difference in the mitochondria-specific MitoSOX^TM^ Red signal was identified using the ‘fluorescence minus background well’ method.

### 2.4. OxLDL Stimulation Elicits Difference in Redox Signature in Patient-Derived ECFCs with or without CAD

**Figure 6:**
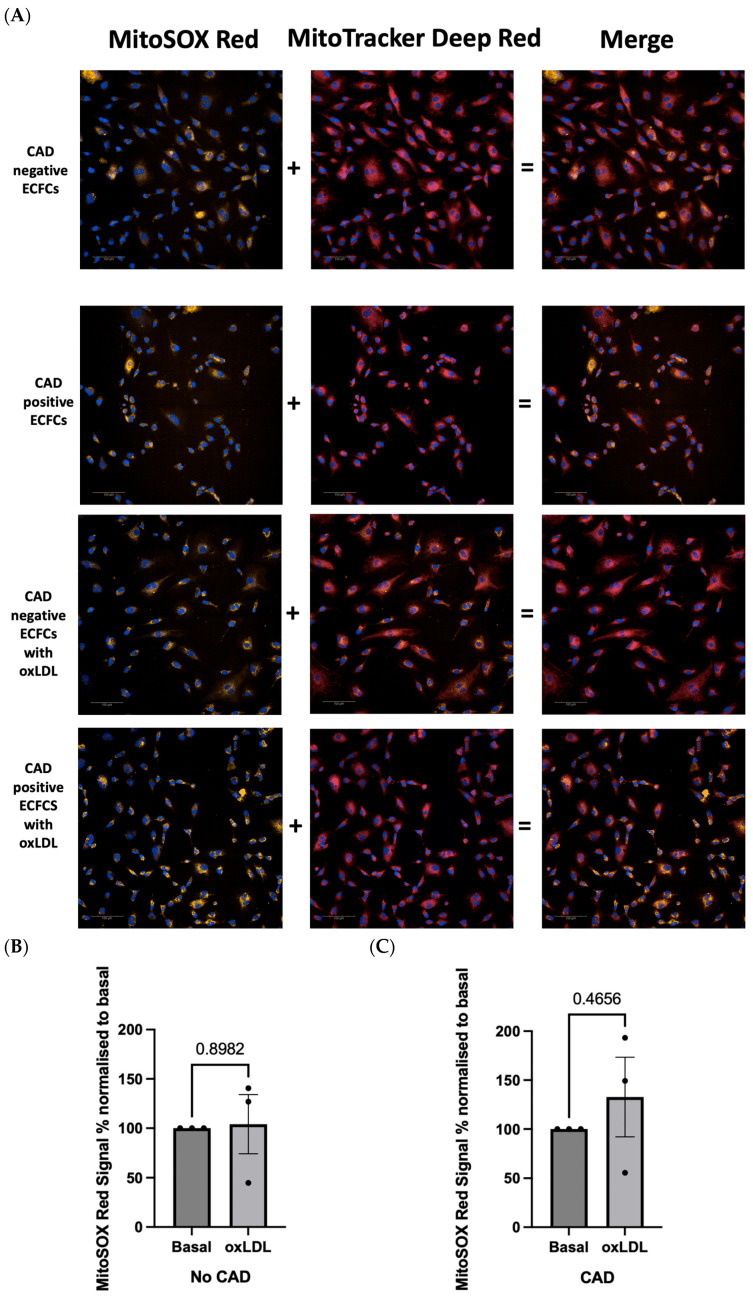

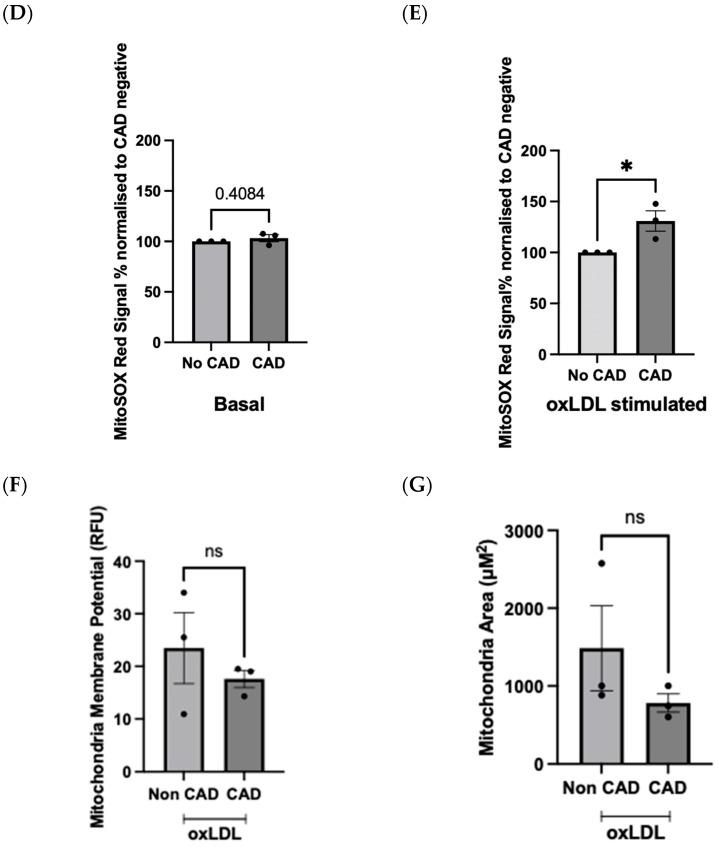
OxLDL stimulation realises differences in redox signature in pooled groups of patient-derived ECFCs with or without CAD. (**A**) Representative confocal images of pooled patient-derived ECFCs with no CAD, pooled patient-derived ECFCs with CAD, oxLDL-stimulated, pooled patient-derived ECFCs with no CAD and oxLDL-stimulated, pooled patient-derived ECFCs with clinically actionable CAD stained with 5 μM MitoSOX^TM^ Red (yellow), 10 nM MitoTracker^TM^ Deep Red (red) and using 5 μM MitoSOX^TM^ Red (yellow), 20 nM MitoTracker^TM^ Deep Red (red), and Hoechst 33342 (blue). Ten fields of view were taken per well. Scale bar = 100 μm. (**B**–**E**) MROS was evaluated in pooled groups of patient-derived ECFCs with/without CAD at basal or oxLDL-stimulated conditions using 5 μM MitoSOX^TM^ Red. (**B**,**C**) MitoSOX^TM^ Red Signal % increase was determined by normalising oxLDL-stimulated groups to basal groups. (**D**,**E**) MitoSOX^TM^ Red Signal % increase was determined by normalising pooled groups of patient-derived ECFCs with CAD to pooled groups of patient-derived ECFCs without CAD. Each condition was performed in duplicate. (**F**) Mitochondrial membrane potential and (**G**) mitochondrial area were evaluated in pooled groups of patient-derived ECFCs with/without CAD in oxLDL-stimulated conditions using 20 nM MitoTracker^TM^ Deep Red. Data are represented as mean ± S.E.M. of three independent experiments (*n* = 3). * *p* < 0.05, ns means non-significant, independent samples *t*-test was performed.

To standardise the platform for drug screening and showcase proof-of-principle, we pooled participants based on CAD status (CAD or no CAD), minimising variation related to individual patients [11]. The CACS percentile allowed for the consideration of age and sex for each participant when aggregating as a group. To evaluate differences in the mROS-specific signal, groups of five patients were pooled together and analysed using our new MitoSOX-Opera Phenix method (Figure 6A). Biobank sample limitations from BioHEART-CT prevented analysis of patient-derived ECFCs at an individual level; however, this is feasible with a sufficient source of PBMCs. Furthermore, certain ECFC lines were passaged whenever feasible and reused for subsequent experiments. The clinical and demographic features of patients used in this study are presented in Table 1.

No difference in the mROS-specific MitoSOX-Opera Phenix fluorescence was observed in pooled ECFCs from No-CAD participants between basal and oxLDL-stimulated states (mean difference: 4.1% *p* = 0.90, *n* = 3) (Figure 6B). In addition, no difference in the MitoSOX-Opera Phenix signal was found between basal and oxLDL-stimulated CAD pooled ECFCs from participants (mean difference 32.73%, *p* = 0.47, *n* = 3) (Figure 6C). No significant basal differences in fluorescence were observed between pooled ECFC samples of participants with and without CAD (*p* = 0.41, *n* = 3) (Figure 6D). However, when both groups were stimulated with oxLDL, pooled ECFCs from participants with CAD demonstrated a 30.89% increase in the MitoSOX-Opera Phenix signal compared to participants without CAD (CI: 3.24–58.5%, *p* < 0.05, *n* = 3) (Figure 6E). No difference was reported in mitochondrial membrane potential or mitochondrial area between CAD ECFCs and non-CAD ECFCs that were treated with oxLDL (Figure 6F,G).

### 2.5. Evaluation of Compounds and Their Effects on Cell Viability in HUVECs

**Figure 7:**
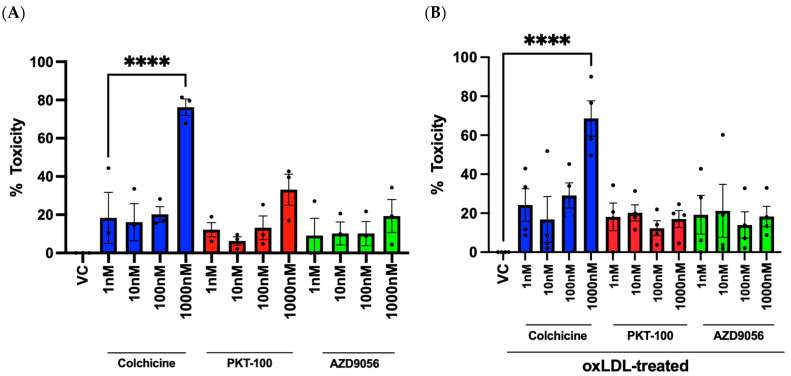
Evaluation of cell viability. HUVECs were treated with compounds and/or oxLDL for 24 h. Cell viability was evaluated using a CellTiter-Glo^®^ kit. Relative cell viability was calculated by normalising cell viability to the vehicle control. Cell viability in HUVECs treated with several concentrations of colchicine, PKT-100, or AZD9056 under (**A**) basal and (**B**) oxLDL-stimulated conditions. Data are represented as mean ± S.E.M. of at least 3 independent experiments. Each condition was performed in triplicate. One-way ANOVA followed by Dunnett’s multiple comparisons test. **** *p* < 0.0001.

To test the drug screening capabilities of the MitoSOX-Opera Phenix method, three candidate compounds, colchicine, AZD9056, and PKT-100, were selected and tested. First, the effect of these compounds on cell viability was evaluated in HUVECs at basal or oxLDL-activated conditions at four concentrations (1 nM, 10 nM, 100 nM, and 1 μM). A 1 μM concentration of colchicine had a detrimental effect on HUVECs under basal and oxLDL-stimulated conditions, reducing cell viability by 76.3% (*p* < 0.0001, *n* = 3) (Figure 7A) and 68.6% (*p* < 0.0001, *n* = 4) (Figure 7B), respectively.

### 2.6. Evaluation of Compounds in Reducing mROS Production in HUVECs Stimulated with oxLDL

**Figure 8:**
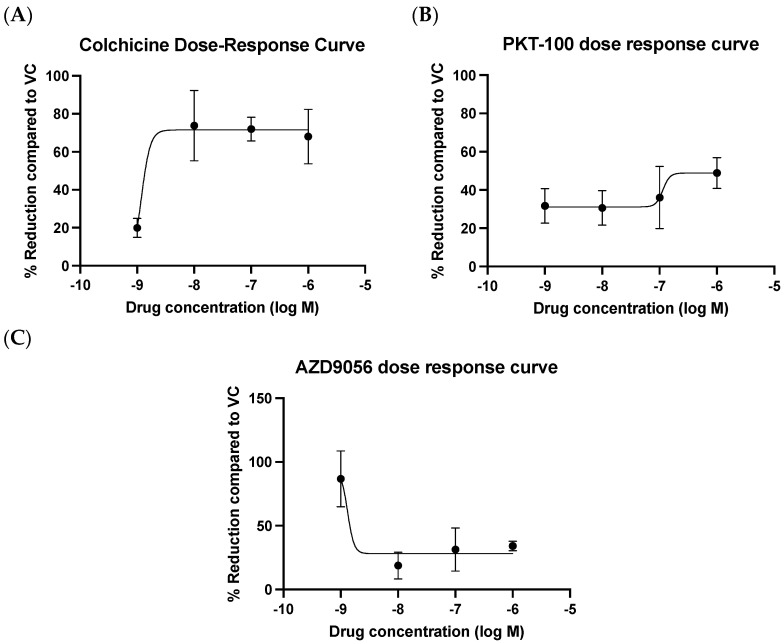
Dose–response curve of (**A**) colchicine, (**B**) PKT-100, and (**C**) AZD9056 on mROS production at different concentrations in oxLDL-stimulated HUVECs. MROS levels were evaluated using 5 μM MitoSOX^TM^ Red at 24 h after compound treatment using a fluorescence microplate reader. The % reduction was determined by normalising MitoSOX^TM^ Red intensity to protein concentration before subsequently normalising to vehicle control. Data are represented as the mean ± S.D. of at least 3 independent experiments. Each condition was performed in triplicate.

The effect of colchicine, PKT-100, and AZD9056 at protecting against mitochondrial redox dysregulation was examined in oxLDL-treated HUVECs. As shown in Figure 8, colchicine reduced the oxLDL-induced MitoSOX^TM^ Red signal. This protective effect was greatest at 1 nM (IC50 = 1.19 nM ± 12.76 nM) (Figure 8A). PKT-100 also reduced the oxLDL-induced MitoSOX^TM^ Red signal (IC50 = 114.20 nM ± 10.72 nM) (Figure 8B), as did AZD9056 (IC50 = 1.32 nM ± 16.21 nM) (Figure 8C). However, as mentioned before, the observed MitoSOX^TM^ Red signal using this method was not exclusively reflective of mROS production; therefore, we aimed to improve the specificity via use of the MitoSOX-Opera Phenix method.

### 2.7. Evaluation of Compounds in Reducing mROS Levels in Pooled Patient-Derived ECFCs with CAD

**Figure 9:**
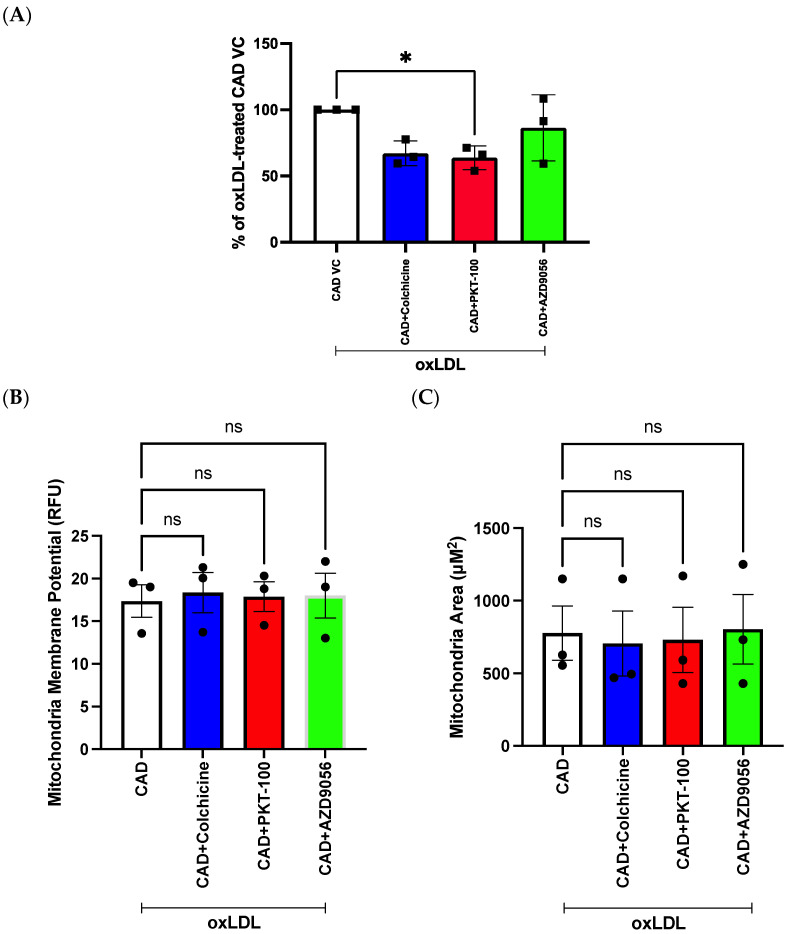
Evaluation of drug candidates on mROS production, mitochondrial membrane potential, and mitochondrial area in pooled patient-derived ECFCs with CAD. (**A**) MROS production was evaluated using 5 μM MitoSOX^TM^ Red at 24 h after 100 nM drug treatment using the Opera Phenix^®^. The % reduction was determined by normalising MitoSOX^TM^ Red intensity of CAD patient-derived ECFCs treated with different compounds to CAD patient-derived ECFCs treated with vehicle control (VC). (**B**) Mitochondrial membrane potential and (**C**) mitochondrial area was evaluated using 20 nM MitoTracker^TM^ Deep Red at 24 h after 100 nM PKT-100 treatment using the Opera Phenix^®^. Data are represented as the mean ± S.D. Results are presented as an average of three independent experiments (*n* = 3). Unpaired *t*-test was performed. * *p* < 0.05, ns means non-significant.

To confirm that the effects of the compounds were indeed specific to mROS levels, we sought to investigate their efficacy (using single concentration 100 nM) in protecting against oxLDL-induced mROS levels in groups of CAD patient-derived ECFCs pooled utilising our highly specific MitoSOX-Opera Phenix method. Preincubation in 100 nM of colchicine, PKT-100, or AZD9056 for 24 h in the presence of oxLDL led to mean reductions of 32.89% (CI: −0.15% to 65.93%, *p* = 0.051), 36.22% (CI: 3.18% to 69.25%, *p* = 0.03), and 13.59% (CI: −19.45% to 46.635, *p* = 0.53), respectively, in oxLDL-induced MitoSOX-Opera Phenix signals (Figure 9A). No significant differences in mitochondrial membrane potential and mitochondrial area were observed in all compounds (Figure 9B and Figure 9C, respectively).

## 3. Discussion

Here, we have demonstrated a novel application of the Opera Phenix^®^ confocal screening platform to investigate the effect of potential therapeutics on mROS production in patient-derived ECFCs. This is a promising drug discovery tool to evaluate the effectiveness of novel compounds urgently needed for the treatment of coronary atherosclerosis. We have developed a novel workflow, the MitoSOX-Opera Phenix method, to accurately identify and measure mROS levels, mitochondrial membrane potential, and mitochondrial size in patient-derived ECFCs.

Whilst we have made inroads to reducing the burden of CAD via the therapeutic targeting of elevated cholesterol and blood pressure, we have had less success in targeting factors involved in the inflammatory response in the vessel wall. This is partly related to the more challenging translational pathway for drugs not acting via traditional risk-factor surrogates. There is an urgent need for human preclinical models of vascular dysfunction and atherosclerosis. The high-throughput confocal assessment of oxLDL-induced MitoSOX^TM^ using the Opera Phenix^®^ platform offers promise.

OxLDL was chosen due to its key role in the pathogenesis of human CAD. It is known to inhibit endothelial nitric oxide synthase activity and production of nitric oxide [18], and to increase ROS production [19] in endothelial cells. In tandem, redox imbalance occurs, leading to oxidative stress and mitochondrial dysfunction in endothelial cells [20]. MitoSOX^TM^-based assays are widely used for the measurement of mROS production due to their simplicity, efficiency, and inexpensiveness [21]. However, some degree of non-specificity is observed and virtually impossible to eliminate using a simple plate reader, generating artefacts which can lead to unreliable detection of mROS production [21,22]. This may have contributed to the higher-fold increase in fluorescence using the microplate reader compared with the Opera Phenix^®^ (Figure 1 and Figure 3), highlighting the benefits of the confocal approach and single-cell sorting capabilities. In addition, other parameters like mitochondrial membrane potential and mitochondrial area need to be accounted for as they can influence the MitoSOX^TM^ signal [12]. However, basic microplate readers cannot be multiplexed with multiple fluorescent probes without some degree of spectral overlap and cannot perform colour compensation [23]. Only instruments such as flow cytometers and high-content-screening (HCS) imaging instruments like the Opera Phenix are enabled for multiplexing several fluorescent probes [24]. However, unlike flow cytometry or confocal microscopy, the Opera Phenix^®^ has a much higher throughput as it can perform automated scans and analyses in a 96-well plate format under cell culture conditions (37 °C, 5% CO_2_). The MitoSOX-Opera Phenix method mitigates non-specific fluorescence by performing automated analysis of high-resolution confocal images allowing precise attribution of the MitoSOX^TM^ signal solely to the mitochondrial region of individual cells (Figure 3). In addition, the Opera Phenix^®^ confocal system offers a unique capability to capture and measure both mitochondrial membrane potential and mitochondrial area using only MitoTracker^TM^ Deep Red. As such, using the MitoSOX^TM^ Red and MitoTracker^TM^ Deep Red together allows for powerful multi-parametric analyses to be performed.

The use of an oxLDL-rich environment was able to elicit important functional differences between ECFCs derived from participants with CAD compared to those without (Figure 5). Using the MitoSOX-Opera Phenix method, a 30.9% increase in mROS production was found in pooled ECFCs from participants with CAD compared to those without CAD, which was only evident in an oxLDL-stimulated environment (Figure 5C). This MitoSOX signal increase was independent of changes in mitochondrial membrane potential and mitochondrial size (Figure 6F,G). This is consistent with findings from our previous study using flow cytometry [11], and suggest that the antioxidant defence system of ECFCs derived from participants without CAD may substantially be higher and is capable of protecting against redox imbalances caused by noxious stimuli like oxLDL which upregulates ROS generation [19]. This supports the potential value of these patient-derived cells to investigate individual susceptibility to CAD in inflammatory environments like oxLDL, and to test the efficacy of novel drugs relevant to human disease.

The potential for our MitoSOX-Opera Phenix method to be a valuable drug discovery tool was demonstrated by testing on a small number of candidate compounds: colchicine, PKT-100, and AZD9056 (Figure 9). Colchicine, a pre-established FDA-approved drug for treating CAD, exhibited a reduction in mROS levels (Figure 9A, *p* = 0.051). Previous studies have demonstrated the protective effects of colchicine at low doses [25,26,27], decreasing cholesterol crystal-induced ROS generation in HUVECs via the AMPK/SIRT1 pathway [27], and more recently driving improved cardiovascular outcomes in clinical trials (0.5 mg/day) [25,26]. Interestingly, we were able to delineate for the first time the antioxidant effect of the P2X7 antagonists in endothelial cells with the compound PKT-100 (Figure 8 and Figure 9). PKT-100 (100 nM) significantly decreased mROS levels by 36.22% in patient-derived ECFCs compared to the vehicle control, independent of changes to mitochondrial membrane potential and mitochondrial size (Figure 9). Previously, PKT-100 was shown to protect against pulmonary hypertension in bleomycin-treated mice [28]. The P2X7 receptor is highly expressed in endothelial cells [29] and activation of this receptor in endothelial cells has been shown to escalate the plaque development process [30,31,32,33,34]. A proposed pathway in which P2X7 affects mROS levels is via the MAPK ERK1/2 and the NADPH oxidase complex pathways [35]. Overall, the proof-of-principle findings from this study support the potential for the high-throughput screening platform to be used for drug development for CAD.

Some limitations of this study should be acknowledged. First, the pooling of patient-derived ECFCs was solely based on CAD severity, specifically CACS percentile, which allowed us to account for age and sex. However, we were unable to consider SMuRFs which themselves are known to contribute to oxidative stress. Furthermore, clinical characteristics collected from participants used in the pooled study indicated that age, hypertension, and statin usage were significant contributors (*p* < 0.05) to differences (Table 1). The rarity and labour-intensive method of culturing these patient-derived ECFCs limited the number of available samples, making it challenging to account for these additional variables, highlighting a key drawback for future implementation. Future studies should include hypertensive and medication-matched controls, which may have confounded differences between the CAD and no-CAD cohorts. Previous findings have also highlighted sex-specific differences in patient-derived ECFCs, with females demonstrating a strong correlation between Gensini score and MitoSOX^TM^ signal [11]. Therefore, the incorporation of sex as a factor in pooling strategies could prove valuable.

In addition, issues with the specificity of MitoSOX^TM^ Red persist. Whilst the MitoSOX-Opera Phenix method goes to great lengths to localise the MitoSOX^TM^ Red signal to the mitochondria, as well as measuring mitochondrial membrane potential and mitochondrial size to ensure that it does not play a role in affecting the MitoSOX^TM^ Red signal, it is unable to differentiate between its two oxidation products: the non-specific ethidium and the specific 2-hydroxyethidium [12,36]. The two products have overlapping fluorescent spectra of the O_2_^−^-specific product, 2-hydroxyethidium (2-HE), that can only be differentiated using liquid-mass spectrometry (LC-MS) [12,36]. Rather, future studies should incorporate more specific mitochondrial probes like MitoNeoD, which has been proven to be specific for O_2_^−^ and overcomes the aforementioned limitations of MitoSOX^TM^ Red [37].

## 4. Materials and Methods

### 4.1. Cell Culture

HUVECs or patient-derived ECFCs were thawed from either −80 °C or liquid nitrogen (LN_2_) storage and maintained under normal cell culture conditions at 37 °C with 5% CO_2_. The cells were cultured in Endothelial Growth Medium-2 (EGM-2) basal media supplemented with the EGM-2 SingleQuot Kit (Lonza, Norwest, Australia), which contained appropriate supplements and growth factors following the manufacturer’s recommendations. The final concentration of foetal bovine serum (FBS) in the culture medium was 2%. Regular passaging of the cells was performed when they reached approximately 80% confluency. For this study, endothelial cells from passages 4 to 6 were utilised.

### 4.2. Study Population and Recruitment

Samples included in this study were from the BioHEART-CT cohort (Australia New Zealand Clinical Trials Registry ANZTR12618001322224, Camperdown, Australia), a longitudinal, prospective, multi-centre cohort study of participants referred for clinically indicated coronary CT angiography [38]. Adults with previously diagnosed or suspected CAD were invited to provide written informed consent for this study. Clinical data is collected via facilitated interview at the time of recruitment and includes demographics, anthropometrics, past medical history, family history, medications, occupational and exposure history, and indication for CCTA.

Twenty-eight participants meeting the CAD endpoints, CAD positive = CACS percentile > 70% and no CAD = 0% CACS percentile, were included in the cell function analysis. Participants with a prior history of myocardial infarction, percutaneous coronary intervention, or coronary artery bypass graft were excluded. Participants included in this analysis were recruited between March 2018 and November 2021.

### 4.3. Imaging Analysis

Coronary CT angiograms (CCTA) were performed using a 256-slice CT scanner following standard clinical protocols [38,39]. In cases where necessary, heart-rate-limiting medications such as beta-blockers or ivabradine were administered orally to optimize heart rate prior to the CCTA procedure. To minimize radiation exposure, the current recommendations [40] for dose reduction were strictly adhered to during the scans. The assessment of coronary artery calcium score (CACS) was conducted prior to CCTA using the Agatston method [41]. This method quantifies the amount of calcified plaque present in the coronary arteries. A CACS percentile adjusted for age and sex was produced for each participant.

### 4.4. PBMC Isolation and ECFC Growth

Peripheral blood samples were collected immediately after the insertion of the peripheral venous cannula required for CCTA. Blood tubes with lithium heparin anticoagulant were utilised for PBMC isolation. Following collection, lithium heparin tube(s) were stored at room temperature until processing. Within four hours of collection, PBMCs were isolated using a standard Ficoll preparation method [42]. The PBMCs were then plated onto T-25 flasks coated with 0.1% gelatin (ThermoFisher Scientific, Waltham, MA, USA) at a density of 2.5 × 10^4^ cells/cm^2^. Endothelial cell growth medium (EGM-2) (Lonza, Basel, Switzerland), supplemented with the EGM-2 SingleQuot Kit and 2% FBS, was used as the culture medium. The flasks containing the plated PBMCs were cultured at 37 °C with 5% CO_2_ for up to 21 days, with regular monitoring of spontaneous ECFC growth. As the ECFCs matured, cell lines were expanded into T-75 flasks and 10 cm^2^ dishes for subsequent analyses. Upon reaching passage 4, the successfully matured ECFCs were cryopreserved in a solution of 10% dimethyl sulfoxide (DMSO) and 90% FBS. The cryopreserved ECFCs were stored in LN_2_ or −80 °C for long-term preservation and future use in downstream experiments and analyses.

### 4.5. System Setup

The experimental protocol employed the Opera Phenix^®^ High-Content Screening System (PerkinElmer, USA), which utilizes three lasers with different wavelengths. The first laser is a blue laser with a wavelength of 405 nm, utilized to identify the cell nuclei using the NucBlue™ Live ReadyProbes™ Reagent (Hoechst 33342) (Invitrogen, Waltham, MA, USA). The second laser is a red laser with a wavelength of 640 nm, used to identify the mitochondrial region using the MitoTracker™ Deep Red FM (Invitrogen, USA). The third laser is a yellow laser with a wavelength of 561 nm, employed to detect mitochondrial superoxide production using the MitoSOX™ Mitochondrial Superoxide Indicators for live-cell imaging (Invitrogen, USA). Throughout the experiments, the samples were maintained at standard cell culture conditions of 5% CO_2_ and 37 °C. Compensation was not necessary as the Opera Phenix^®^ system’s spinning disk technology, with increased pinhole-to-pinhole spacing, effectively reduces out-of-focus noise and fluorescence cross-talk. This feature ensures improved image quality and minimizes any potential interference between fluorescence channels.

### 4.6. Opera Phenix Image Analysis

All raw fluorescence measurements were analysed using the Harmony^®^ High-Content Imaging and Analysis Software (PerkinElmer, USA, version 4.8). The software performed a series of calculations to determine the MitoSOX^TM^ Red signal for each well measurement. First, individual cell nuclei were identified using the Hoechst 33342 channel. Next, the software captured the mitochondrial region surrounding each nucleus using the MitoTracker^TM^ Deep Red channel. Finally, the MitoSOX^TM^ Red signal within the mitochondrial region was measured. To calculate the MitoSOX^TM^ Red signal, the software summed the total MitoSOX^TM^ Red signal detected in the mitochondrial region across all cells and then averaged it by the number of cells analysed. This process allowed for quantitation of the MitoSOX^TM^ Red signal specifically within the mitochondria of the cells. To eliminate any background fluorescence, the ‘sliding parabola’ algorithm was implemented [43]. This algorithm effectively reduced noise and enhanced the accuracy of the MitoSOX^TM^ Red signal measurements. For each experimental condition, the MitoSOX^TM^ Red signal was averaged across duplicate or triplicate wells to obtain the final value. To facilitate comparisons across independent experiments, the MitoSOX^TM^ Red results were presented as a percentage increase relative to the positive control/negative control. The MitoTracker^TM^ Deep Red signal was averaged across duplicate or triplicate wells to obtain a final value. The MitoTracker^TM^ Deep Red results were presented as relative fluorescence units (RFU). The mitochondrial area was calculated using the Harmony^®^ software and presented as μM^2^.

### 4.7. Cell Viability Assay

HUVECs were seeded at a density of 1 × 10^4^ cells/well in 96-well white microplates (Greiner, Monroe, NC, USA) and incubated for 24 h under normal cell culture conditions. Following the initial incubation period, the cells were treated with compounds and/or 31.25 μM oxLDL (Invitrogen, USA), which were diluted in complete medium, for an additional 24 h in the same normal cell culture conditions. To assess cell viability, a CellTiter-Glo^®^ Luminescent Cell Viability Assay (Promega, Madison, WI, USA) was prepared according to the manufacturer’s instructions. After the treatment period, the cells were washed twice with warm 1× PBS to remove any residual compounds or media. Then, 50 μL of the CellTiter-Glo^®^ reagent was added to each well. The contents of the wells were mixed for two minutes on an orbital shaker to induce cell lysis and ensure complete release of intracellular ATP. The microplates were incubated for 10 min at room temperature to establish a stable luminescent signal, allowing the luminescent intensity to reflect the ATP content and, consequently, the cell viability. The luminescent signal was subsequently recorded using a Synergy™ 2 Microplate reader (BioTek, Winooski, MT, USA) equipped for luminescence detection. All experiments were performed in triplicate.

### 4.8. Microplate Reader mROS Production Assay

HUVECs were seeded at a density of 1 × 10^4^ cells/well in 96-well black microplates (Corning, Corning, NY, USA) and allowed to adhere for 24 h under normal cell culture conditions. Following the initial incubation, the cells were treated with compounds and/or 31.25 μM oxLDL for a duration of 24 h under the same normal cell culture conditions. To assess mROS production, cells were washed twice with warm 1× PBS to remove any residual media or compounds. Subsequently, 5 μM MitoSOX^TM^ Red dye (ThermoFisher, USA) was added to each well and incubated for 25 min under normal cell culture conditions. Cells were washed twice with warm 1× PBS again and resuspended in 100 μL of Flurobrite™ DMEM (Gibco, Grand Island, NY, USA). The fluorescence signal of MitoSOX^TM^ Red was measured using a microplate reader (FLUOstar^®^ Omega, BMG Labtech, Ortenberg, Germany) with excitation at 490 nm (Ex bandwidth: 10 nm) and emission at 590 nm (Em bandwidth: 10 nm). To minimize the effects of uneven cell distribution, orbital averaging was employed during the readings. After the MitoSOX^TM^ Red fluorescence measurement, the cells were placed on ice, washed once with ice-cold 1× PBS, and subsequently lysed with RIPA buffer. The protein concentration was determined using a BCA assay. This allowed for the normalization of the MitoSOX^TM^ Red signal to the protein concentration, providing a more accurate representation of mROS levels in relation to cellular protein content. All experiments were performed in triplicate.

### 4.9. Opera Phenix mROS Production and Drug Screening Assay

Initially, patient-derived ECFCs were pooled and seeded at a density of 0.5 × 10^4^ cells/well in PhenoPlate^TM^ 96-well plates and incubated for 24 h. Following this, the cells were treated with 31.25 μM oxLDL and/or compounds, which were diluted in complete medium, for a duration of 24 h under normal cell culture conditions. Prior to analysis, the cells were washed twice with warm 1× PBS. For mitochondrial analysis, a combination of fluorescent dyes was used. A cocktail containing 5 μM MitoSOX^TM^ Red, 20 nM MitoTracker^TM^ Deep Red, and NucBlue^TM^ Live ReadyProbe^TM^ was prepared in Flurobrite™ DMEM supplemented with 5% HIFBS. This cocktail was added to each well (100 μL/well) and incubated for 25 min under normal cell culture conditions. Subsequently, the cells were washed twice with FluroBrite^TM^ DMEM to remove any excess dye and then resuspended in FluroBrite^TM^ DMEM for live-cell imaging. All experiments were performed in duplicate. Pooled CAD-positive patient-derived ECFCS were included as a positive control whilst pooled no-CAD patient-derived ECFCs were included as a negative control, while MitoTEMPO, an mROS scavenger, served as a negative drug control in the experiments.

### 4.10. Statistical Analyses

Data are expressed as the mean ± SD for the indicated number of experiments. For statistical analysis between two groups, an unpaired student’s *t*-test was performed. For comparison between several groups, one-way ANOVA was performed. For multiple comparisons, one-way ANOVA was performed, followed by Dunnett’s multiple comparisons test. Categorical variables were presented as frequencies and percentages, while continuous variables were reported as means ± standard deviations for normally distributed data, and medians with interquartile ranges for non-normally distributed data. To compare categorical variables, Pearson’s Chi-squared test was utilised, while Student’s *t*-tests were employed for continuous variables. All statistical analyses were performed using GraphPad Prism, version 10.0.0., San Diego, CA, USA, and Jamovi, version 2.3., Sydney, NSW, Australia.

## 5. Conclusions

This study successfully establishes a high-throughput screening method for evaluating mROS levels using the Opera Phenix^®^ High-Content System. The use of patient-derived ECFCs and the Opera Phenix^®^ allows for the multi-parameter characterization of phenotypic differences under various conditions. Stimulation with oxLDL significantly upregulated mROS production in pooled groups of patient-derived ECFCs with CAD independent of significant changes to mitochondrial membrane potential and mitochondrial size. We have also identified, using this system, the P2X7 receptor antagonist compounds, PKT-100, as a novel agent for reducing excess mROS levels in endothelial cells. Further studies should account for additional variables and stratifications to gain a more comprehensive understanding of mitochondrial function in the context of CAD. In addition, the utilisation of more specific probes such as MitoNeoD should also be considered. By addressing these limitations and exploring other promising compounds, we can pave the way for the pre-clinical development of targeted therapies aimed at alleviating excess mROS levels and improving patient outcomes in CAD and related conditions.

## Figures and Tables

**Table 1:** Clinical characteristics of the population of patients.

Characteristic	Whole Cohort (*n* = 28)	CAD (*n* = 14)	No CAD (*n* = 14)	*p*-Value
Age, median (IQR)	54.5 (15.3)	59 (16.3)	49.1 (11.0)	0.007
Female, *n* (%)	13 (46.4)	4 (28.6)	9 (64.3)	0.06
Hypertension, *n* (%)	9 (32.1)	8 (57.1)	1 (7.1)	0.005
Diabetes mellitus, *n* (%)	2 (7.1)	1 (7.1)	1 (7.1)	1.000
Hypercholesteremia, *n* (%)	14 (50)	8 (57.1)	6 (42.9)	0.45
Significant smoking history, *n* (%)	5 (17.9)	2 (14.3)	3 (21.4)	0.62
Current smoker, *n* (%)	0 (0)	0 (0)	0 (0)	N/A
BMI, mean, (SD)	26.6 (5.3)	25.8 (4.0)	27.4 (6.4)	0.44
Significant family history CAD, *n* (%)	14 (50)	7 (50)	7 (50)	1.0
SMuRFs, *n*, (%)	19 (67.9)	10 (71.4)	9 (64.3)	0.69
No SMuRFs, *n* (%)	9 (32.1)	4 (28.6)	5 (35.7)	0.69
Coronary artery calcium score—median, (25th, 75th percentile)	1.1 (0, 286.51)	298.9 (177.7, 499.2)	0 (0, 0)	<0.001
Coronary artery calcium score percentile—median (25th, 75th percentile)	35.5 (0, 77.8)	78.5 (75.25, 87.8)	0 (0, 0)	<0.001
Medication use:				
Anti-coagulant—*n* (%)	4 (14.3)	2 (14.3)	2 (14.3)	1.0
Anti-platelet agent—*n* (%)	6 (21.4)	2 (14.3)	4 (28.6)	0.36
Statin—*n* (%)	8 (28.6)	7 (50)	1 (7.1)	0.012
Beta-blocker—*n* (%)	24 (85.7)	12 (85.7)	12 (85.7)	1.0
ACE/ARB agent—*n* (%)	8 (28.6)	6 (42.3)	2 (14.3)	0.09
Anti-inflammatory agent—*n* (%)	24 (85.7)	11 (78.6)	13 (92.9)	0.28

## Data Availability

The data presented in this study are available on request from the corresponding author.
